# Dissociating task, semantic, and response conflict in cognitive aging: evidence from a low-cognitive control Stroop paradigm

**DOI:** 10.1007/s00426-026-02399-y

**Published:** 2026-10-16

**Authors:** Eldad Keha, Maria Augustinova, Ludovic Ferrand, Eyal Kalanthroff

**Affiliations:** 1 Department of Psychology, The Hebrew University of Jerusalem, Mt. Scopus, Jerusalem, 91905 Israel; 2 Department of Social and Health Sciences, Bar-Ilan University, Ramat Gan, Israel; 3 Department of Psychological and Brain Sciences, Washington University in Saint Louis, St. Louis, Missouri USA; 4 Université de Rouen Normandie, Centre de Recherche sur les Fonctionnements et Dysfonctionnements Psychologiques, Mont-Saint-Aignan, France; 5 Université Clermont Auvergne, CNRS, LAPSCO, Clermont-Ferrand, France

## Abstract

Cognitive aging is often associated with increased Stroop interference, but it remains unclear whether this reflects a broad decline in inhibitory control or selective impairments in specific conflict-resolution mechanisms. The present study examined age-related differences in task conflict, semantic conflict, and response conflict using a low-cognitive-control Stroop paradigm designed to amplify conflict effects. Younger and older adults completed a manual color-identification task including neutral shapes, neutral words, color-associated words, non-response-set incongruent color words, and response-set incongruent color words. This design allowed us to dissociate response conflict, two components of semantic conflict, and task conflict within a single paradigm. Older adults showed larger response conflict than younger adults, along with modest age-related increases in both semantic relevance and semantic relatedness. In contrast, task conflict did not differ between age groups. These results suggest that age-related increases in Stroop interference are not best explained by a single global inhibitory deficit. Rather, under the present manual-response, reduced-control conditions, older adults showed greater semantic and response-level conflict, whereas task conflict remained comparable across age groups. This dissociation is consistent with a more fine-grained account of cognitive control in aging.

**Supplementary Information:**

The online version contains supplementary material available at https://doi.org/10.1007/s00426-026-02399-y.

## Public significance statements

Everyday life often requires selecting relevant information or actions while ignoring competing information, such as following a conversation in a distracting environment. In a manual-response Stroop task designed to reduce control over word processing, older adults showed greater response conflict and modestly greater semantic conflict than younger adults, whereas task conflict did not differ between the groups. These findings suggest that aging may affect some forms of interference more than others and motivate future research examining how specific conflict processes relate to functioning in distracting everyday situations.

**Supplementary Information:**

The online version contains supplementary material available at https://doi.org/10.1007/s00426-026-02399-y.

Cognitive control refers to a set of mental operations that support goal-directed behavior, particularly in contexts involving interference or competing responses, and is therefore essential for navigating complex environments (Miyake et al., 2000). Cognitive control evolves across the lifespan and declines with healthy aging. These age-related changes have been well documented, with older adults typically exhibiting reduced performance on tasks that demand high levels of regulatory processing and executive functioning (e.g., Hasher & Zacks, 1988; Maldonado et al., 2020; Salthouse et al., 2003). These declines are particularly evident in core components of cognitive control, such as inhibition, working memory updating, and task switching (see, e.g., Braver & Barch, 2002; Miyake et al., 2000; Treitz et al., 2007). Consequently, older adults consistently show larger interference effects than younger adults in tasks requiring the suppression of prepotent or irrelevant responses, including the Stroop task, flanker task, stop-signal task, and various working memory paradigms (see, e.g., Guay et al., 2026; Jaroslawska & Rhodes, 2019; Nicosia et al., 2021; Rey-Mermet & Gade, 2018). Because these differences remain even after controlling for age-related differences in processing speed, they have been interpreted as evidence of a general decline in inhibitory control, reflecting difficulties in resisting distraction and disengaging from no longer relevant information (e.g., Gazzaley & D’Esposito, 2007; Hasher & Zacks, 1988; Spieler et al., 1996). These behavioral patterns are consistent with broader neurobiological changes associated with aging, including age-related declines in the structural integrity of brain regions and connections that support cognitive control, which have been linked to poorer performance on tasks involving inhibition, switching, and working memory (Colcombe et al., 2005; Hoagey et al., 2019; Kennedy & Raz, 2009; Raz & Rodrigue, 2006; Ziegler et al., 2010). The goal of the present study was to further examine age-related differences in cognitive control in the Stroop task by assessing whether healthy aging differentially affects distinct sources of interference.

## Age-related changes in Stroop performance

A widely used paradigm for assessing cognitive control is the color-word Stroop task (Stroop, 1935), in which participants are instructed to identify the ink color of written words while ignoring the words’ semantic content. The task typically involves three primary conditions: incongruent trials, in which the word meaning and ink color mismatch (e.g., the word “RED” printed in blue); congruent trials, in which the word meaning matches the ink color (e.g., “RED” printed in red); and neutral trials, which involve either non-color words (e.g., “TABLE”) or non-lexical stimuli such as letter strings (e.g., “XXXX”) or symbol strings (e.g., “####”). Stroop effects, taken as evidence of interference from the irrelevant reading dimension (Kane & Engle, 2003; MacLeod, 1991), are characterized by slower and less accurate responses on incongruent trials compared with neutral or congruent trials. Cognitive control determines the magnitude of these differences, with more efficient control yielding smaller Stroop effects. Accordingly, older adults typically show larger Stroop effects than younger adults, even after controlling for differences in processing speed (e.g., Aschenbrenner & Balota, 2015; Bugg et al., 2007; Davidson et al., 2003; Jackson & Balota, 2013; Nicosia et al., 2021, for a meta-analysis; Spieler et al., 1996; Treitz et al., 2007; Verhaeghen & De Meersman, 1998). However, Stroop effects have increasingly been conceptualized as reflecting two distinct types of conflict: information conflict and task conflict (Kalanthroff et al., 2018; Keha & Kalanthroff, 2023, 2025; MacLeod & MacDonald, 2000; Parris et al., 2022). Information conflict arises when two pieces of information contradict each other (e.g., the word “BLUE” printed in red). This form of interference is typically measured by comparing reaction times (RTs) between incongruent and neutral word trials, a difference commonly referred to as the Stroop interference effect. Task conflict, by contrast, reflects competition between the task of naming the ink color and the automatic tendency to read the word, regardless of whether the word meaning is congruent or incongruent (see Littman et al., 2019; Parris et al., 2023, 2025). This type of conflict is often assessed using the facilitation effect, comparing neutral nonword and congruent trials (Kalanthroff et al., 2013; Keha & Kalanthroff, 2023), or more accurately using the word interference effect (Augustinova et al., 2019; Ferrand et al., 2020; Keha & Bugg, 2026; Keha & Kalanthroff, 2025, 2026; Kinoshita et al., 2018), which compares neutral word trials to neutral nonword trials (e.g., “TABLE” vs. “⬛⬛⬛⬛” presented in red ink; see Table 1).

**Table 1:** Operational definitions of different types of conflicts in the current experiment

Name of marker	Contrast	Example
Response conflict	Response set incongruent – Non-response set incongruent	BLUE_red_ – GRAY_red_
Semantic conflict:		
Semantic relevance	Non-response set incongruent – Color-associated words	GRAY_red_ – BANANA_red_
Semantic relatedness	Color-associated words – Neutral words	BANANA_red_ – AREA_red_
Task conflict	Neutral words – Neutral shapes	AREA_red_ – ⬛⬛⬛⬛_red_

Note. The table summarizes the four conflict markers that were used in the current study, including the name of each conflict type, the contrast used to compute it, and an illustrative example. In the examples, the word is capitalized, and the ink color is indicated using a subscript

Importantly, consistent with the idea that age-related decline in cognitive control may be specific rather than general, Keha et al. (2025) provided direct evidence for a dissociation between these conflict types in aging. Older adults exhibited greater information conflict than younger adults, as reflected in larger Stroop interference effects. In contrast, task conflict was similar across age groups, as indicated by comparable word interference effects, suggesting that aging may spare the ability to resolve task-level competition while having a detrimental effect on the ability to resolve information-level competition. Given the limited evidence, the first goal of the present study was to replicate this dissociative pattern. The second goal was to address the still unresolved question of why older adults exhibit greater information conflict than younger adults by providing a more precise decomposition of information conflict into its semantic and response components. 

### Age-related changes in semantic and response components of information conflict

The question of why older adults exhibit greater information conflict than younger adults echoes recent conceptual and methodological developments that have enabled researchers to more precisely disentangle the components of information conflict in the Stroop task (Augustinova & Ferrand, 2014; Augustinova et al., 2019; Shichel & Tzelgov, 2018). These developments originate from the so-called semantic gradient proposed by Klein (1964; see also Levin & Tzelgov, 2016; Sharma & McKenna, 1998), which examines whether the strength of the association between word meaning and color determines the magnitude of interference in the Stroop task. Klein demonstrated a semantic gradient such that standard incongruent trials (e.g., the word “BLUE” presented in red, when blue is part of the response set) produce more interference than non-response set incongruent trials (e.g., the word “BROWN” presented in red, when brown is not part of the response set). These trials, in turn, produce more interference than color-associated words (e.g., “BANANA” presented in red), which themselves produce more interference than neutral words (e.g., “TABLE” presented in red).

Building on Klein’s work, Li and Bosman (1996) examined whether this semantic gradient changes with age. This question is theoretically important because it goes beyond whether older adults exhibit greater Stroop interference (a marker of information conflict) and instead addresses which component of interference is most sensitive to aging. Specifically, examining the semantic gradient can reveal whether older adults are disproportionately affected by semantically related distractors, suggesting less efficient control over spreading semantic activation. Interestingly, Li and Bosman’s findings showed no age-related differences in the semantic gradient: both younger and older adults exhibited increasing interference as semantic relatedness increased, with the exception of standard incongruent trials, which produced disproportionately larger interference in older adults. On this basis, Augustinova et al. (2018) argued that reduced cognitive control in healthy aging selectively affects the ability to resolve response conflict (i.e., conflict arising when the activated word meaning elicits a competing response; e.g., Zysset et al., 2001) but not semantic conflict (i.e., conflict arising when the activated word meaning is related to the ink color; Augustinova & Ferrand, 2014; Augustinova et al., 2015; Parris et al., 2022, for a review; Shichel & Tzelgov, 2018). Consistent with this account, Augustinova et al. (2018) found no age-related differences in the magnitude of semantic conflict induced by color-associated words (e.g., “BANANA” presented in red) but substantial age-related differences in the response conflict generated by standard incongruent trials (e.g., “BLUE” presented in red, when blue is part of the response set).

While the evidence for age-related differences in response conflict appears convincing, attempts to replicate these findings using a different variant of the Stroop task, namely, the two-to-one Stroop paradigm, have yielded a different pattern of results. Introduced by De Houwer (2003), this variant maps two (out of four) colors onto the same response keys, thereby creating same-response incongruent trials, in which the word and ink color mismatch semantically but converge on the same response, and different-response incongruent trials, in which they mismatch both semantically and at the response level. Within this framework, same-response trials are taken to index semantic conflict with minimal response competition, whereas the additional slowing observed for different-response relative to same-response trials indexes response conflict. Using this paradigm, Burca et al. (2022) provided evidence that semantic (not response) conflict increases in older adults. Furthermore, Burca et al. (2025) showed that older adults exhibit larger magnitudes of both response and semantic conflict, with the semantic component being disproportionately increased. Thus, findings from the two-to-one paradigm suggest that age-related Stroop deficits may not be confined to response conflict alone but may also involve impaired control over semantic conflict. Moreover, Burca et al. (2025) further examined the processes underlying age-related differences in Stroop interference, building on Jackson and Balota’s (2013) distinction between reduced task-set quality and greater degradation of the task set over time. They compared short and long response-stimulus intervals (RSIs), reasoning that if older adults have greater difficulty maintaining the color-naming task set, age differences in Stroop interference should increase as the interval becomes longer. However, the effect of RSI was comparable across age groups, suggesting relatively preserved task-set maintenance in older adults. Instead, decomposition of Stroop interference revealed larger semantic and response conflict in older adults, with the age difference particularly pronounced for semantic conflict. Thus, their findings suggest that age-related differences are better characterized by reduced task-set quality, particularly in controlling semantic interference, than by greater degradation of the task set over time. Taken together, these findings challenge the view of response conflict as the sole or dominant source of age-related differences in Stroop effects and provide strong evidence that the effects of healthy aging are perhaps more complex, such that semantic and response conflict can be affected differently by healthy aging.

The literature reviewed above reveals inconsistent findings regarding age-related changes in the control of semantic and response conflict. One possible explanation for these discrepancies lies in the degree of cognitive control required by the task context. Specifically, it is possible that, in some studies, proactive control masked conflict effects, thereby obscuring age-related differences (Kalanthroff et al., 2013; Keha et al., 2025). However, conflict effects can be modulated by experimental manipulations that reduce cognitive control, such as increasing the proportion of nonword trials or decreasing the frequency of conflict trials (Bugg, 2014; Bugg et al. 2011; Goldfarb and Henik 2007; Keha and Kalanthroff 2023, 2025, 2026; Keha et al. 2024a, b; Shichel and Tzelgov 2018; Tzelgov et al. 1992). In other words, under such low-control conditions, conflict effects become more apparent. Accordingly, the reduced cognitive control condition implemented in the present study was expected to provide a more stringent test of age-related differences in semantic and response conflict (see Table 1).

### The present study

Consistent with the view that age-related decline in cognitive control is not general, but rather that some components of cognitive control are relatively preserved in older adults, the present study examined age-related changes in the magnitude of task, semantic, and response conflict. These changes were investigated using a low-cognitive control manual^1^ Stroop paradigm, which provides a particularly suitable context for isolating the contributions of different types of conflict to the Stroop effect, as well as age-related differences in these contributions (Kalanthroff et al., 2013; Keha et al., 2025). The magnitude of task conflict was operationalized as the contrast between neutral word trials and neutral shape trials (see Table 1). Based on Keha et al. (2025), we expected no age-related differences in task-set activation (see also Li & Bosman, 1996), such that the magnitude of task conflict would be comparable in younger and older adults. Unlike Keha et al. (2025), who examined overall information conflict, the present study further decomposed this type of conflict into response and semantic components to assess whether healthy aging differentially affects the control of these two forms of interference. Response conflict was indexed by the difference between response set incongruent and non-response set incongruent trials (Li & Bosman, 1996; Parris et al., 2019, 2021; Quétard et al., 2023; Sharma & McKenna, 1998). Based on prior findings, we expected response conflict to be larger in older than in younger adults (Augustinova et al., 2018; Burca et al., 2025; Li & Bosman, 1996).

Following Li and Bosman (1996; see also Klein, 1964; Levin & Tzelgov, 2016; Quétard et al., 2023; Sharma & McKenna, 1998), semantic conflict was examined at two levels (see Table 1). First, semantic relevance (see Sharma & McKenna, 1998, for introducing the distinction between semantic relevance vs. relatedness) was indexed by the difference between non-response set incongruent trials and color-associated word trials (see also Hasshim & Parris, 2018; Hasshim et al., 2019; Klein, 1964; Lamers et al., 2010; Quétard et al., 2023; Sharma & McKenna, 1998). Second, semantic relatedness (see Sharma & McKenna, 1998, for using this term) was indexed by the difference between color-associated word trials and neutral word trials (see also Augustinova & Ferrand, 2014; Augustinova et al., 2010; Augustinova et al., 2019; Ferrand & Augustinova, 2014; Ferrand et al., 2020; Kinoshita et al., 2018; Neely & Kahan, 2001). Although semantic relatedness and semantic relevance have been proposed to reflect overlapping semantic processes (Kinoshita et al., 2018), they capture experimentally distinguishable sources of semantic interference. Indeed, semantic relatedness concerns the semantic relationship between the distractor and the target, whereas semantic relevance concerns the relevance of the distractor to the task at hand (La Heij, 1988; La Heij et al., 1990). These components have been experimentally distinguished in previous Stroop and Stroop-like paradigms, although they have also been proposed to arise within a common processing locus (Sharma & McKenna, 1998). We therefore considered it important to examine these components separately in the context of cognitive aging. Examining these components separately allowed us to determine whether age-related differences were confined to one source of semantic interference or emerged across both.

## Method

### Participants

A total of 160 participants took part in the experiment in return for £2. The experiment was conducted online via the Prolific platform. All procedures involving human participants were approved by the Ethics Committee of Achva Academic College (Approval No. Achva-0113) and were conducted in accordance with the Declaration of Helsinki. All participants reported having no history of mild cognitive impairment, dyslexia, attention deficit hyperactivity disorder (ADHD), dementia, or any neurological disorders. In addition, all participants reported having normal or corrected-to-normal vision and no color blindness. Young adults had to be at least 18 years old but not over 40. Older adults had to be at least 65. Two participants from the older-adult group and two participants from the younger-adult group were excluded because their accuracy rates were extremely low, falling more than 3 SDs below their group mean. One additional younger adult was excluded because their mean RT was extremely fast, falling more than 3 SDs below the younger-adult group mean. Therefore, the analyzed sample included 78 older adults and 77 younger adults (see Table 2 for demographics).

**Table 2:** Participant characteristics by age group

	Older adults (*N* = 78)	Younger adults (*N* = 77)		Statistical differences between groups
Male/Female	37/41	38/39		χ^2^(1) = 0.006, *p =* .938
Right/Left	69/9	64/13		χ^2^(1) = 0.523, *p* = .470
Age	70.5 (5.06) [65–87]		29.8 (5.00) [20–40]	*t*(153) = 50.224, *p* < .001
Education	14.9 (2.81) [10–23]		15.6 (3.57) [4–23]	*t*(153) = 1.186, *p =* .238

Note. Values are mean (SD) with ranges in brackets. Sex and handedness are reported as counts. Participants reporting color blindness, dementia, mild cognitive impairment, dyslexia, ADHD, or neurological disorders were excluded based on self-report

### Sample size consideration

An a priori power analysis was conducted using G*Power 3.1.9.7 (Faul et al., 2007). We calculated the desired sample size to detect the 5 × 2 mixed-model ANOVA interaction with stimulus type (neutral shapes, neutral words, color-associated words, non-response set incongruent, and response set incongruent) as a within-subject factor and age group (younger adults vs. older adults) as a between-subject factor. We based our calculations on a similar interaction that was reported in Augustinova et al. (2018). To ensure that our sample size was sufficient to detect age-related differences for each of the conflict types (see Table 1), we based our power analysis on the smallest effect size that was reported in Augustinova et al. (2018), for the stimulus type × age group interaction ($\:{{\upeta\:}}_{p}^{2}$ = 0.031, which is equivalent to Cohen’s f = 0.179). Using a conservative significance criterion (α = 0.05, two-tailed) and targeting high power (1 − β = 0.99), the analysis indicated a required total sample of *N* = 150, corresponding to 75 participants per age group. Anticipating exclusions, we aimed to recruit 80 participants per group (*N* = 160).

### Materials and stimuli

Stimuli consisted of English words or geometric shapes presented in one of four ink colors: red, green, blue, or yellow. There were five stimulus categories: (1) neutral shapes: neutral geometric shapes made of four characters (e.g., “♦♦♦♦”, “▲▲▲▲”), (2) neutral words: neutral words unrelated to color (e.g., the word “AREA” presented in red), (3) color-associated words: words that are semantically related to colors and appeared in an incongruent color (e.g., the word “GRASS” presented in blue), (4) Non-response set incongruent: color words that were not part of the response set in the experiment and appeared in an incongruent color (e.g., the word “PINK” presented in yellow), and (5) response set incongruent: color words that were part of the response set in the experiment and appeared in an incongruent color (e.g., the word “RED” presented in blue). All stimuli were displayed in 22-point Arial font at the center of a black screen, and each stimulus in the experiment could appear in only one ink color (see Table 3).

**Table 3:** Stimulus categories and items used in the Stroop task

Neutral shapes	Neutral words	Color-associated words	Non-response set incongruent	Response set incongruent	Presented color
⬛⬛⬛⬛	AREA	BANANA	GRAY	BLUE	RED
●●●●	MEET	SKY	PURPLE	YELLOW	GREEN
♦♦♦♦	FACT	GRASS	BROWN	RED	BLUE
▲▲▲▲	LINE	FIRE	PINK	GREEN	YELLOW

Note. Each stimulus appeared in one ink color only. Color-associated words were semantically related to a specific color but were not color names. Non-response set incongruent items were color words not included in the response set, and response set incongruent items were color words included in the response set

### Procedure

All participants provided informed consent before beginning the experiment. Participants began with a practice block of 20 trials to familiarize themselves with the mapping between colors and response keys. Responses were made using the keys “C,” “V,” “B,” and “N,” which corresponded to the colors red, green, blue, and yellow, respectively. Participants were instructed to place four fingers of their dominant hand on the response keys and to respond as quickly and accurately as possible. In the practice phase, participants identified the color of a large asterisk presented at the center of the screen until a response was made. Feedback (“correct” or “incorrect”) was provided following each practice trial. Following the practice block, participants completed the experimental phase, which consisted of four blocks of 192 trials each. Within each block, there were 128 neutral shape trials, 16 neutral words, 16 color-associated words, 16 non-response set incongruent, and 16 response set incongruent trials. The order of trials was fully randomized within each block, with no restrictions imposed on the sequencing of trial types. Each trial began with a 1,000 ms fixation cross, followed by the stimulus, which was presented for 2,000 ms or until a response was detected. A 500-ms blank screen separated trials.

### Statistical analysis

All statistical analyses were conducted in R version 4.4.1 (R Core Team, 2024). Reaction-time (RT) analyses were restricted to correct-response trials. For each participant, trials with RTs more than 3 SDs above or below that participant’s mean within each stimulus type were excluded, resulting in the removal of 1.64% of trials. To control for generalized age-related slowing, data was standardized within participant by subtracting that participant’s mean RT and dividing by their RT standard deviation, following Jackson and Balota (2013; see also Faust et al., 1999). The Z-scores were converted from the trimmed data with correct responses only and 3 SDs above or below the participant’s mean within each stimulus type condition.

The primary RT analysis was a 5 × 2 mixed-model ANOVA, with stimulus type (neutral shapes, neutral words, color-associated words, non-response set incongruent, and response set incongruent) as a within-subject factor and age group (younger adults vs. older adults) as a between-subjects factor. Planned comparisons examined age-related differences in the four conflict indices defined a priori: response conflict (response set incongruent – non-response set incongruent), semantic relevance (non-response set incongruent – color-associated words), semantic relatedness (color-associated words – neutral words), and task conflict (neutral words – neutral shapes). P-values for these comparisons were corrected for multiple comparisons using the Benjamini–Hochberg procedure. Effect sizes are reported as partial η² for ANOVA effects and Cohen’s d for between-group comparisons. Accuracy rates were analyzed using the same 5 × 2 mixed-model ANOVA and the same planned conflict comparisons. Accuracy proportions were transformed using an arcsine square-root transformation to improve normality prior to inferential analysis. Results from the primary analyses of z-transformed RTs and arcsine-transformed accuracy rates are reported in the main text, whereas the corresponding analyses of raw RTs and untransformed accuracy rates, together with the complete post hoc comparisons, are provided in Online Resource 1. The datasets used for these analyses are available on the Open Science Framework at https://osf.io/nf4eg/?view_only=0d39acc490d44be4b4601302a2e0bb25.

## Results

### Reaction time (RT)

A main effect was found for stimulus type, *F*(4, 612) = 441.417, *p* < .001, $\:{{\upeta\:}}_{p}^{2}$ = 0.743 (see Online Resource 1 for all possible post hoc comparisons of this main effect). In addition, a main effect was found for age group, *F*(1, 153) = 19.125, *p* < .001, $\:{{\upeta\:}}_{p}^{2}$ = 0.111. Importantly, a significant two-way interaction was found between stimulus type and age group, *F*(4, 612) = 13.358, *p* < .001, $\:{{\upeta\:}}_{p}^{2}\:$= 0.080. To further examine this interaction, we tested age-related differences in each of the conflicts of interest, including response conflict (response set incongruent – non-response set incongruent), semantic relevance (non-response set incongruent – color-associated words), semantic relatedness (color-associated words – neutral words) and task conflict (neutral words – neutral shapes). We corrected for multiple comparisons using the Benjamini–Hochberg correction. Larger effects in older adults were evident for response conflict, *t*(153) = 2.049, *p* = .047, Cohen’s d = 0.329, for both components of semantic conflict: semantic relevance, *t*(153) = 2.145, *p* = .047, Cohen’s d = 0.345, and semantic relatedness, *t*(153) = 2.075, *p* = .047, Cohen’s d = 0.333, whereas task conflict did not differ between younger and older adults, *t*(153) = 0.707, *p* = .481, Cohen’s d = 0.114.

### Accuracy

Accuracy rates were transformed using an arcsine square-root transformation to improve normality prior to analysis. A main effect was found for stimulus type, *F*(4, 612) = 17.328, *p* < .001, $\:{{\upeta\:}}_{p}^{2}$ = 0.102 (see Online Resource 1 for all possible post hoc comparisons of this main effect). No main effect was found for age group, *F*(1, 153) = 1.230, *p* = .269, $\:{{\upeta\:}}_{p}^{2}\:$= 0.008. Finally, no significant two-way interaction was found between stimulus type and age group, *F*(4, 612) = 1.991, *p* = .094, $\:{{\upeta\:}}_{p}^{2}$ = 0.013. The planned comparison analysis, however, showed that older adults exhibited a larger response-conflict accuracy cost than younger adults, *t*(153) = 2.028, *p* = .044, Cohen’s *d* = 0.326. No age group differences were found for semantic relevance, *t*(153) = 0.113, *p* = .910, Cohen’s *d* = 0.018; semantic relatedness, *t*(153) = 0.145, *p* = .885, Cohen’s *d* = 0.023; or task conflict, *t*(153) = 0.328, *p* = .743, Cohen’s *d* = 0.053. (Table 4)

**Fig. 1:**
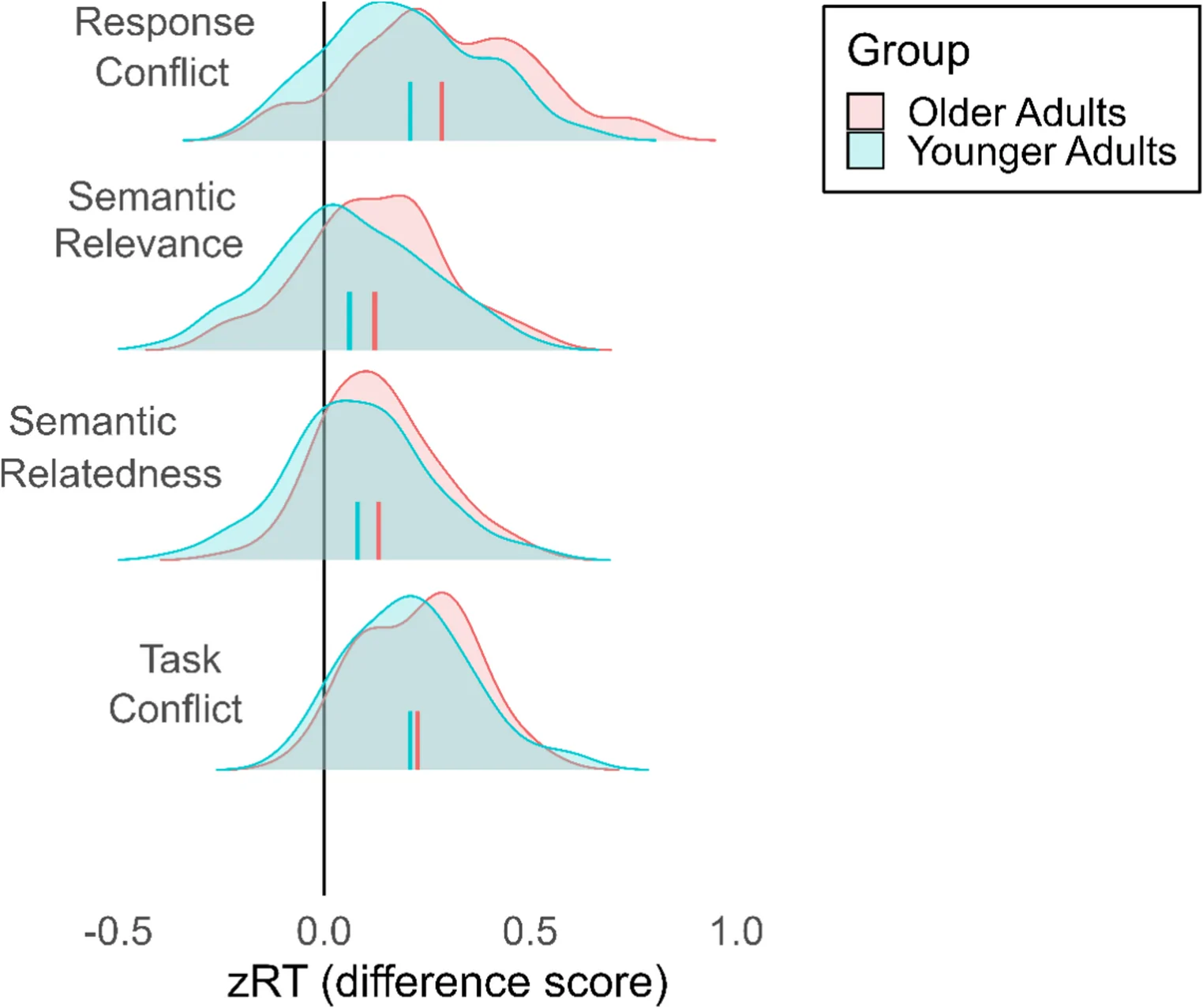
Distributions of conflict effects in reaction time by age group. Note. Each panel shows the kernel density distribution of participants’ z-scored reaction time (zRT) difference scores for a given conflict type, separately for older and younger adults. The vertical black line marks 0, indicating no effect. Positive values indicate greater interference (slower responding in the first condition than the second) and negative values indicate the reverse. Short vertical colored markers within each panel indicate the group mean difference score for that conflict type. Difference scores were computed as: response conflict = response set incongruent minus non-response set incongruent; semantic relevance = non-response set incongruent minus color-associated words; semantic relatedness = color-associated words minus neutral words; task conflict = neutral words minus neutral shapes

**Table 4:** Descriptive statistics and conflict indices by age group for reaction time and accuracy

	RT(Z-scores)		RT(Raw scores)		ACC(Arcsine)		ACC(Raw scores)	
	Older adults	Younger adults	Older adults	Younger adults	Older adults	Younger adults	Older adults	Younger adults
*Neutral shapes*	-0.193 (0.007)	-0.163 (0.005)	722 ms (11 ms)	626 ms (11 ms)	1.386(0.011)	1.364(0.009)	95.8%(0.5%)	95.2%(0.4%)
*Neutral words*	0.038 (0.012)	0.052 (0.014)	778 ms (12 ms)	672 ms (13 ms)	1.392(0.015)	1.365(0.013)	95.2%(0.6%)	94.6%(0.5%)
*Color-associated words*	0.173 (0.015)	0.133 (0.014)	806 ms (11 ms)	688 ms (13 ms)	1.385(0.015)	1.360(0.011)	95.1%(0.7%)	94.8%(0.4%)
*Non-response set incongruent*	0.305 (0.021)	0.198 (0.018)	843 ms (14 ms)	708 ms (14 ms)	1.393(0.016)	1.366(0.014)	95.1%(0.7%)	94.6%(0.6%)
*Response set incongruent*	0.589 (0.029)	0.409 (0.024)	920 ms (15 ms)	762 ms (16 ms)	1.317(0.018)	1.328(0.013)	91.8%(1.1%)	93.2%(0.6%)
			Older adults	Younger adults	Age effect (RT)	Older adults	Younger adults	Age effect (ACC)
*Response conflict* (Response set incongruent – Non-response set incongruent)			0.284zs [77 ms]	0.211zs [55 ms]	0.073zs* [*22 ms*]	-0.076 [-3.3%]	-0.038 [-1.5%]	-0.038* [-1.9%]
*Semantic conflict:*								
*Semantic relevance* (Non-response set incongruent – Color-associated words)			0.132zs [37 ms]	0.066zs [20 ms]	0.066zs* [*17 ms*]	0.008 [0.1%]	0.006 [-0.2%]	0.002^ns^ [0.3%]
*Semantic relatedness* (Color-associated words – Neutral words)			0.135zs [28 ms]	0.081zs [16 ms]	0.054zs* [*12 ms*]	-0.007 [-0.1%]	-0.004 [0.2%]	-0.002^ns^ [-0.3%]
*Task Conflict* (Neutral words – Neutral shapes)			0.231zs [56 ms]	0.214zs [46 ms]	0.017zs^ns^ [*11 ms*]	0.005 [-0.7%]	0.001 [-0.6%]	0.004^ns^ [-0.1%]

Note. The upper section presents condition-level descriptive statistics separately for older and younger adults. For each condition, reaction times (RTs) are presented as participant-level z-scored means and raw RT means, with standard errors in parentheses. Accuracy is presented as arcsine square-root transformed means and raw accuracy percentages, with standard errors in parentheses. The lower section presents the four conflict indices of interest: response conflict, semantic relevance, semantic relatedness, and task conflict. For each RT conflict index, the z-scored effect is shown first, followed by the corresponding raw RT difference in brackets. For each accuracy conflict index, the arcsine-transformed effect is shown first, followed by the corresponding raw accuracy difference in percentage points in brackets. Age effects reflect the older-adult effect minus the younger-adult effect for each contrast. Negative accuracy effects indicate lower accuracy in the first condition of the contrast relative to the second condition. Follow-up *p*-values were corrected using the Benjamini–Hochberg procedure

*RT* reaction time, *ms*  milliseconds, *zs* z-score, *ns* not significant

* = *p* < .05

## Discussion

Building on the idea that age-related decline in cognitive control is not uniform, the present study examined age-related changes in the control of task, semantic, and response conflict. Task conflict was operationalized as the contrast between neutral word and neutral shape trials; semantic conflict was examined via both semantic relevance (indexed by the difference between non-response set incongruent and color-associated word trials) and semantic relatedness (indexed by the difference between color-associated and neutral word trials); and response conflict was indexed by the difference between response set and non-response set incongruent trials. Combining these contrasts provides a comprehensive assessment of age-related effects in the Stroop task, capturing multiple sources of interference within a single design (see Augustinova et al., 2018; Burca et al., 2022, 2025; Keha et al., 2025). To our knowledge, no previous study has jointly examined age-related differences in task, semantic, and response conflict, allowing for a direct comparison of their relative sensitivity to aging. Critically, this investigation was conducted under reduced cognitive control conditions (via a high proportion of neutral shape trials), a context known to unmask conflict effects that may be attenuated under strong proactive control (Bugg, 2014). This approach therefore provided a more stringent test of whether and where age-related differences emerge in Stroop performance. Consistent with this reasoning, even after controlling for general slowing (via z-transformed reaction times), older adults exhibited larger interference effects for semantic and response-related contrasts, whereas task conflict did not differ by age. Although the conflict contrasts differed in their overall RT costs, the observed pattern of age effects does not support a simple difficulty-based account since task conflict produced a larger RT cost than either semantic conflict effect but did not show an age-related increase, whereas both smaller semantic conflict effects did. This dissociation between age-related increases in semantic and response-related conflict and the absence of an age-related increase in task conflict challenges a general inhibitory deficit account of aging, which predicts broadly reduced ability to suppress irrelevant activation (Hasher & Zacks, 1988; Hasher et al., 1991; Lustig et al., 2007). Taken together, these findings are consistent with recent accounts questioning a uniform age-related inhibition deficit (Burca et al., 2022; Ileri-Tayar et al., 2025; Keha et al., 2025; Rey-Mermet & Gade, 2018). Instead, the present results point to a more selective vulnerability in resolving semantic interference and response competition, while the ability to suppress the irrelevant word-reading task appears relatively preserved.

### Age-related differences at the response level

As mentioned above, age-related differences at the response level were particularly robust. In line with previous studies (Burca et al., 2025; Killikelly & Szűcs, 2013; Li & Bosman, 1996), older adults exhibited larger response conflict in reaction time. The accuracy data showed a descriptively similar pattern, although this pattern should be interpreted cautiously because the omnibus Age group × Stimulus Type interaction was not significant. Importantly, the present study employed a different approach to indexing response conflict than the two-to-one paradigm, by comparing response set incongruent trials with non-response set incongruent trials. This contrast isolates the cost associated with distractors that activate response-eligible competitors while holding semantic mismatch constant (but see Parris et al., 2022; Roelofs, 2003, for a discussion of this issue). This methodological distinction is important because, as noted by Burca et al. (2025), conclusions about specific Stroop components can depend on how contrasts are defined. In particular, the two-to-one paradigm assumes that same-response trials reflect pure semantic conflict, but these trials may also involve response facilitation. As a result, apparent age differences in semantic conflict could partly reflect greater facilitation in younger adults rather than greater conflict in older adults. By contrast, the present approach provides a more direct estimate of response competition. The convergence of findings across methods strengthens the conclusion that aging is reliably associated with reduced efficiency in resolving response-level competition.

This pattern aligns with prior evidence suggesting that aging disproportionately affects late-stage conflict resolution processes. For instance, Spieler et al. (1996; see also Jackson & Balota, 2013) showed that age-related increases in Stroop interference are driven by a subset of trials with particularly prolonged reaction times, reflected in a heavier right tail of the RT distribution for incongruent trials. Together, these findings support the interpretation that aging primarily impairs the ability to suppress or override response-eligible distractors, consistent with the present results.

### Age-related differences at the semantic level

The present study also provides modest evidence for age-related differences in semantic conflict. Older adults exhibited larger effects for both semantic relevance and semantic relatedness. This absence of a dissociation between semantic relatedness and semantic relevance across age groups is theoretically informative at least at two levels. First, although these components capture experimentally distinguishable sources of semantic interference, their similar age-related pattern suggests that aging does not selectively affect one component relative to the other. This finding is compatible with the possibility that they rely, at least in part, on overlapping processing stages or mechanisms (Kinoshita et al., 2018; Sharma & McKenna, 1998), while not implying that the two components are functionally identical. Second, the age-related difference at this specific component is also consistent with results from the two-to-one paradigm (Burca et al., 2022, 2025), but contrasts with earlier studies reporting no age-related differences in semantic conflict (Augustinova et al., 2018; Li & Bosman, 1996).

Several factors may account for these discrepancies. First, in line with our reasoning, the low-control context used in the present study likely increased the behavioral expression of semantic conflict, which is typically small in magnitude (Kinoshita et al., 2018; Parris et al., 2022), thereby allowing age-related differences to emerge. Second, the present study employed a larger sample than prior work, increasing statistical power to detect subtle effects. Third, the use of z-transformed reaction times helped control for general slowing. By contrast, proportional transformations used in earlier studies (e.g., Augustinova et al., 2018; Li & Bosman, 1996) may unintentionally bias results in favor of older adults, potentially masking true age-related differences (Burca et al., 2022, for a re-analysis of Augustinova et al.’s data consistent with this idea; Hedge et al., 2018). Finally, response modality may also play a role. Unlike prior studies using vocal responses, the present study employed manual responses, which require an additional stimulus-response translation step (Glaser & Glaser, 1982). This increased reliance on controlled response selection may amplify the impact of semantic interference, particularly in older adults. Supporting this interpretation, Doose and Feyereisen (2004) reported a robust Age × Response modality interaction, with older adults showing faster vocal than manual responding in tasks that require mapping a stimulus decision onto a response. This response modality effect has been linked to the greater demands imposed by arbitrary stimulus-response mappings on older adults, which may make semantic distractors more disruptive in the manual task, thereby increasing measured semantic conflicts with key presses compared with a vocal response. Accordingly, the present findings regarding age-related differences in semantic conflict should be interpreted within the boundary conditions of the manual response Stroop task and should not be assumed to generalize directly to vocal responding. To examine this option, future studies, therefore, can test whether there are age-related differences in different types of conflicts as a function of response type.

An additional consideration for the semantic level effects concerns the different patterns observed in RT and accuracy for the semantic relevance and semantic relatedness effects. These semantic conflict effects were expressed in RT but produced little to no accuracy cost in either age group. This pattern is consistent with previous manual Stroop studies, in which semantic conflict is often more readily detected in RT than in accuracy, whereas accuracy effects are more consistently concentrated at the response-conflict level (Augustinova et al., 2019; Burca et al., 2022; Kinoshita et al., 2018). Thus, accuracy may be relatively insensitive to the small semantic conflict effects observed in the present paradigm. Importantly, although overall performance may reflect some degree of speed-accuracy trade-off, such a trade-off is unlikely to fully account for the age-related discrepancy between RT and accuracy patterns in semantic conflict. If such a trade-off explained the results, one would expect smaller conflict effects for the older group that was more accurate. Instead, response conflict showed the same age-related pattern in both measures, with older adults exhibiting larger interference in both RT and accuracy, and all the other conflict effects showed no age-related difference in accuracy. Thus, the dissociation between the semantic RT and accuracy findings is more consistent with the limited sensitivity of accuracy to semantic conflict in a manual Stroop task than with a speed–accuracy trade-off.

### Age-related differences at the task level

Despite the reduced-control context, no age-related differences were observed in task conflict. These findings therefore provide a higher-powered replication of Keha et al. (2025) in an online sample. They also speak to the task-set maintenance account proposed by Jackson and Balota (2013). Specifically, the absence of an Age × Congruency × RSI interaction in their work suggests that task-set maintenance is relatively preserved in aging. Instead, age-related differences in Stroop performance appear to arise primarily at later stages of processing, once word-related interference has been activated. This interpretation is further supported by Burca et al. (2025), who likewise found no evidence for age-related deficits in task-set maintenance, instead attributing observed effects to reduced efficiency in interference control. In sum, no age-related differences in task-set activation reported in the present study provide additional evidence in favor of preservation of the ability of older adults to maintain the color-naming task set consistently over time (but see Parris et al., 2023, for alternative interpretation of task conflict).

### Limitations

The current study has several limitations. One limitation concerns the interpretation of the low-cognitive control manipulation. The high proportion of neutral-shape trials was used as an established means of creating a context in which cognitive control over word processing is reduced, thereby increasing the behavioral expression of the different conflict components. Although proportion-based manipulations are commonly interpreted as reflecting adjustments in cognitive control, the precise mechanisms underlying adaptation to such contexts remain debated (see Parris et al., 2025 for a special issue and Braem et al., 2019 for a review). For example, alternative accounts propose that proportion-based effects may partly reflect learned stimulus–response contingencies rather than adjustments in cognitive control (Schmidt & Besner, 2008; Schmidt, 2019). From an input-driven selective-attention perspective, control adjustment effects may instead arise from stimulus salience and correlations within the stimulus set, with the predominance of neutral-shape trials potentially reducing vigilance toward the relatively infrequent word dimension and producing a baseline shift in attentional allocation (Algom & Chajut, 2019). Importantly, the present study was not designed to determine whether younger and older adults differ in how they adjust their control settings to changes in task context, nor to isolate the specific processes underlying such adjustments. Accordingly, the present findings should be interpreted as demonstrating age-related differences in semantic and response conflict, rather than in a deficit in cognitive control adjustments. Future studies should directly manipulate control context across age groups and examine whether younger and older adults differ in how they adapt cognitive control at different levels of processing, and whether such adaptations contribute to the age-related differences observed in the individual components of Stroop interference.

Another limitation concerns the online nature of data collection and the representativeness of the Prolific sample. Although Prolific employs participant-verification and platform-level quality-control procedures (e.g., verification of email addresses, phone numbers, and photo identification; checks for duplicate accounts, inconsistencies in location information, and VPN or proxy use) and has shown relatively high response validity compared with other opt-in online samples (Stagnaro et al., 2026), online testing provides less control over participants’ testing environments, devices, and attentiveness, potentially introducing additional variability in response-time measures. In addition, no separate questions assessing participants’ motivation, attentiveness, or effort were included, limiting our ability to evaluate these factors directly. Moreover, older adults recruited through an opt-in online platform may be more digitally literate and educated than the broader older population. The online design also limited our ability to administer standardized cognitive screening, such as the MoCA or the MMSE. Future studies should therefore examine the generalizability of these findings in more diverse older samples tested under controlled laboratory conditions and with standardized cognitive screening.

### Implications

Beyond the Stroop-specific mechanisms discussed above, the present findings contribute to broader accounts of executive functioning in aging. Executive control is increasingly viewed as a collection of partially separable processes rather than a unitary ability, and meta-analytic and psychometric evidence indicates that age-related changes vary considerably across executive functions and measures (Maldonado et al., 2020; Rey-Mermet & Gade, 2018; Rey-Mermet et al., 2018). The present study extends this componential perspective by demonstrating that such differentiation can be observed across distinct sources of interference within a single experimental paradigm. This has an important methodological implication for cognitive-aging research. Aggregate measures of interference may conceal meaningful differences in the processes contributing to performance, and conclusions about a general decline in “inhibition” may therefore depend on which component of interference is being measured. This point is consistent with previous arguments that the conventional Stroop effect is a composite phenomenon whose constituent processes should be distinguished rather than treated as a single index of executive control (Augustinova et al., 2019; Parris et al., 2022). The potential implications for everyday functioning should be considered more cautiously, because the present study did not directly assess functional outcomes. Nevertheless, many everyday situations require individuals to select goal-relevant information or actions while competing information is simultaneously available. One potentially relevant domain is communication. Robinson et al. (2025) found that older adults experienced greater semantic interference during propositional speech production in the presence of meaningful distractors, suggesting that competition from task-irrelevant semantic information may contribute to the increased effort required for communication in distracting environments. More broadly, executive functioning has been associated with performance on instrumental activities of daily living, including managing finances and medications, shopping, household activities, and transportation (Vaughan & Giovanello, 2010). Importantly, however, these relationships differ across executive processes, reinforcing the need to identify the specific control processes that contribute to everyday functioning rather than relying on a global measure of executive ability. A similar issue may be relevant to pathological aging. Stroop performance has been used to characterize cognitive impairment, including in Alzheimer’s disease (Hutchison et al., 2010; Guarino et al., 2019; Spieler et al., 1996), but the composite nature of Stroop interference raises the possibility that examining its constituent components could provide a more precise characterization of which control processes are affected. Future studies should therefore directly examine whether semantic, response, and task-conflict measures differentially predict everyday functioning or distinguish healthy from pathological cognitive aging. 

## Conclusions

Overall, the present findings indicate that age-related increases in Stroop interference are not well captured by a single, global inhibitory deficit account. Rather, the results suggest selective age-related differences across different subcomponents. Under the present manual-response, reduced-control context, response conflict was larger in older adults, and modest age-related differences were also observed for both components of semantic conflict, whereas task conflict remained comparable across age groups. These findings are consistent with a more fine-grained account of cognitive aging in which different components of conflict may be differentially affected by aging. Although the semantic age effects were relatively small and should therefore be interpreted cautiously, their emergence across both semantic measures provides preliminary evidence for age-related differences in semantic interference under the present task conditions.

## Supplementary Information

Below is the link to the electronic supplementary material.

Supplementary Material 1 (DOCX 27.8 KB)

## Data Availability

The datasets generated and analyzed during the current study are available on the Open Science Framework at https://osf.io/nf4eg/?view_only=0d39acc490d44be4b4601302a2e0bb25.

## Footnotes

1 We chose to employ the manual rather than the vocal version of the Stroop task primarily because it provides a clearly defined and standardized response set, which is particularly appropriate for the present decomposition of response conflict based on whether distractor words correspond to response-eligible colors. Manual responding is also well established in aging studies separating response, semantic, and task components of Stroop interference (e.g., Burca et al., 2022, 2025; Keha et al., 2025). In addition, manual response recordings were necessary to avoid the additional challenges associated with recording and classifying vocal responses online.
