# Supplementary material for: Dissociating task, semantic, and response conflict in cognitive aging: evidence from a low-cognitive control Stroop paradigm
Source: Psychol Res. 2026 Oct 16;90(6):186. doi: 10.1007/s00426-026-02399-y (PMC13675143; doi:10.1007/s00426-026-02399-y)
Supplement: Supplementary file 1 [file 426_2026_2399_MOESM1_ESM.docx]

**Supplementary Materials**

**Reaction Time (RT)**

We carried out a 5 × 2 mixed-model ANOVA on raw RT data, with stimulus-type (neutral shapes, neutral words, color-associated words, non-response set incongruent, and response set incongruent) as a within-subject factor and age-group (younger adults vs. older adults) as a between-subject factor. As in the main analysis for Z-scores, only correct responses were included, and trials that were 3 SDs below or above each participant’s mean RT within each stimulus-type condition were removed.

A main effect was found for stimulus type, *F*(4, 612) = 377.296, *p* < .001, $\eta_{p}^{2}$ = .711. Pairwise comparisons corrected using the Benjamini-Hochberg procedure indicated that all stimulus types differed significantly from one another (all *ps* < .001). A main effect was also found for age-group, *F*(1, 153) = 48.712, *p* < .001, $\eta_{p}^{2}$ = .241, such that younger adults responded faster than older adults. Importantly, a significant two-way interaction was found between stimulus-type and age-group, *F*(4, 612) = 14.659, *p* < .001, $\eta_{p}^{2}$ = .087. To further examine this interaction, we tested age-related differences in each of the conflicts of interest, including response conflict (response set incongruent – non-response set incongruent), semantic relevance (non-response set incongruent – color-associated words), semantic relatedness (color-associated words – neutral words), and task conflict (neutral words – neutral shapes). Larger effects in older adults were evident for response conflict, t(153) = 2.439, p = .016, semantic relevance, t(153) = 2.050, p = .042, and semantic relatedness, t(153) = 2.046, p = .042. Task conflict did not differ between younger and older adults, t(153) = 1.781, p = .077.

**Accuracy (ACC)**

We carried out the same 5 × 2 mixed-model ANOVA on accuracy rates, with stimulus-type as a within-subject factor and age-group as a between-subject factor. Accuracy was analyzed as subject-level accuracy proportions for each stimulus-type condition.

A main effect was found for stimulus type, *F*(4, 612) = 20.951, *p* < .001, $\eta_{p}^{2}$= .120. Pairwise comparisons corrected using the Benjamini-Hochberg procedure indicated that accuracy was significantly lower for response set incongruent trials than for neutral shapes, *t*(153) = 6.372, *p* < .001, color-associated words, *t*(153) = 5.650, *p* < .001, non-response set incongruent trials, *t*(153) = 5.222, *p* < .001, and neutral words, *t*(153) = 4.965, *p* < .001. Accuracy was also higher for neutral shapes than for neutral words, *t*(153) = 2.559, *p* = .023, color-associated words, *t*(153) = 2.415, *p* = .028, and non-response set incongruent trials, *t*(153) = 2.292, *p* = .033. No significant differences were found among neutral words, color-associated words, and non-response set incongruent trials, all *ps* = .940. No main effect was found for age-group, *F*(1, 153) = 0.030, *p* = .863, $\eta_{p}^{2}$ = .001. However, a significant two-way interaction was found between stimulus-type and age-group, *F*(4, 612) = 2.642, *p* = .033, $\eta_{p}^{2}$ = .017. To further examine this interaction, we tested age-related differences in the same conflict effects used in the RT analysis. A significant age-related difference was found for response conflict, t(153) = 2.028, p = .044, reflecting a larger accuracy cost in older adults than in younger adults. No age-related differences were found for semantic relevance, t(153) = 0.531, p = .596, semantic relatedness, t(153) = 0.594, p = .553, or task conflict, t(153) = -0.197, p = .844.

**Table S1**

| **Comparison** | **Estimate** | **SE** | **t** | **p** |
| --- | --- | --- | --- | --- |
| Response set incongruent – non-response set incongruent | 0.247 | 0.018 | 13.895 | < .001 |
| Response set incongruent – color-associated words | 0.346 | 0.019 | 18.290 | < .001 |
| Response set incongruent – neutral shapes | 0.676 | 0.022 | 30.464 | < .001 |
| Response set incongruent – neutral words | 0.454 | 0.020 | 22.672 | < .001 |
| Non-response set incongruent – color-associated words | 0.099 | 0.015 | 6.398 | < .001 |
| Non-response set incongruent – neutral shapes | 0.429 | 0.017 | 24.783 | < .001 |
| Non-response set incongruent – neutral words | 0.207 | 0.016 | 13.221 | < .001 |
| Color-associated words – neutral shapes | 0.330 | 0.013 | 24.792 | < .001 |
| Color-associated words – neutral words | 0.108 | 0.013 | 8.350 | < .001 |
| Neutral shapes – neutral words | -0.223 | 0.012 | -18.863 | < .001 |

***Post-hoc comparisons (ZRT) for the Main Effect of Stimulus-type***

**Table S2**

***Post-hoc Comparisons (ZRT) between stimulus-type Conditions, Separately for Younger and Older Adults***

| **Group** | **Comparison** | **Estimate** | **SE** | **t** | **p** |
| --- | --- | --- | --- | --- | --- |
| **Younger**  **Adults** | Response set incongruent - non-response set incongruent | 0.209 | 0.025 | 8.309 | < .001 |
|  | Response set incongruent - color-associated words | 0.271 | 0.026 | 10.243 | < .001 |
|  | Response set incongruent - neutral shapes | 0.562 | 0.031 | 18.155 | < .001 |
|  | Response set incongruent - neutral words | 0.352 | 0.028 | 12.605 | < .001 |
|  | Non-response set incongruent - color-associated words | 0.062 | 0.021 | 3.003 | .003 |
|  | Non-response set incongruent - neutral shapes | 0.353 | 0.024 | 14.971 | < .001 |
|  | Non-response set incongruent - neutral words | 0.143 | 0.021 | 6.709 | < .001 |
|  | Color-associated words - neutral shapes | 0.291 | 0.018 | 16.099 | < .001 |
|  | Color-associated words - neutral words | 0.081 | 0.018 | 4.564 | < .001 |
|  | Neutral shapes - neutral words | -0.209 | 0.016 | -12.775 | < .001 |
| **Older Adults** | Response set incongruent - non-response set incongruent | 0.286 | 0.025 | 11.421 | < .001 |
|  | Response set incongruent - color-associated words | 0.408 | 0.026 | 15.545 | < .001 |
|  | Response set incongruent - neutral shapes | 0.768 | 0.031 | 24.981 | < .001 |
|  | Response set incongruent - neutral words | 0.541 | 0.028 | 19.483 | < .001 |
|  | Non-response set incongruent - color-associated words | 0.123 | 0.020 | 6.013 | < .001 |
|  | Non-response set incongruent - neutral shapes | 0.482 | 0.023 | 20.612 | < .001 |
|  | Non-response set incongruent - neutral words | 0.255 | 0.021 | 12.048 | < .001 |
|  | Color-associated words - neutral shapes | 0.359 | 0.018 | 20.029 | < .001 |
|  | Color-associated words - neutral words | 0.133 | 0.018 | 7.480 | < .001 |
|  | Neutral shapes - neutral words | -0.227 | 0.016 | -13.933 | < .001 |

**Ex-Gaussian Analysis
*Method***
To explore whether age differences were expressed in different portions of the reaction-time distribution, ex-Gaussian distributions were fitted separately to each participant's correct, trimmed reaction times in each of the five stimulus-type conditions using the exGAUS distribution in GAMLSS. The fitted parameters were μ (the location of the Gaussian component), σ (the spread of the Gaussian component), and τ (the mean and standard deviation of the exponential component, reflecting the distribution's right tail). A minimum of 30 usable trials was required to fit a participant-by-condition cell. This threshold was a permissive computational criterion and should not be interpreted as indicating that 30 trials provide stable individual parameter estimates. Of the 155 participants available for this analysis, 150 had valid, converged estimates in all five stimulus-type conditions and were retained (73 younger adults and 77 older adults). In total, 770 of 775 participant-by-condition models converged successfully. Across the four non-shape conditions, participants contributed an average of 59.6 usable trials per participant per condition (SD = 3.06). Because the estimates for the non-shape conditions were therefore based on only about 60 observations per cell, particularly the σ and τ estimates may be less stable than estimates derived from larger trial samples. The ex-Gaussian analyses should consequently be regarded as exploratory.

***Results***
Separate 5 (stimulus type) × 2 (age group) mixed ANOVAs were conducted for μ, σ, and τ, with stimulus type as a within-subject factor and age group as a between-subjects factor. Greenhouse-Geisser corrections were applied to effects involving stimulus type. For μ, significant effects were obtained for age group, *F*(1, 148) = 53.70, *p* < .001, $\eta_{p}^{2}$ = .266, stimulus type, *F*(2.80, 414) = 87.20, *p* < .001, $\eta_{p}^{2}$= .371, and their interaction, *F*(2.80, 414) = 8.81, *p* < .001, $\eta_{p}^{2}$= .056. For σ, the effects of age group, *F*(1, 148) = 11.80, *p* < .001, $\eta_{p}^{2}$= .074, stimulus type, *F*(3.12, 462) = 57.30, *p* < .001, $\eta_{p}^{2}$= .279, and their interaction, *F*(3.12, 462) = 3.74, *p* = .010, $\eta_{p}^{2}$= .025, were also significant. For τ, stimulus type was significant, *F*(3.40, 503) = 17.50, *p* < .001, $\eta_{p}^{2}$= .106, whereas neither age group, *F*(1, 148) = 0.85, *p* = .358, $\eta_{p}^{2}$= .006, nor the interaction, *F*(3.40, 503) = 1.02, *p* = .388, $\eta_{p}^{2}$ = .007, was significant.

The four planned age-group differences in response conflict, semantic relevance, semantic relatedness, and task conflict were examined separately for each ex-Gaussian parameter. For μ, response conflict was significantly larger among older adults (M = 68.6 ms) than younger adults (M = 33.5 ms), t(148) = 2.37, p = .019, Cohen’s d = 0.39. The age-group difference was not significant for semantic relevance (older: M = 23.7 ms; younger: M = 7.04 ms), t(148) = 1.58, p = .115, Cohen’s d = 0.26; semantic relatedness (older: M = 27.7 ms; younger: M = 10.1 ms), t(148) = 1.56, p = .121, Cohen’s d = 0.26; or task conflict (older: M = 26.9 ms; younger: M = 35.0 ms), t(148) = −0.93, p = .352, Cohen’s d = 0.15.

For σ, none of the four planned age-group differences was significant. This was the case for response conflict (older: M = 40.4 ms; younger: M = 25.8 ms), t(148) = 1.62, p = .107, Cohen’s d = 0.27; semantic relevance (older: M = 13.6 ms; younger: M = 9.96 ms), t(148) = 0.58, p = .566, Cohen’s d = 0.09; semantic relatedness (older: M = 9.66 ms; younger: M = −1.62 ms), t(148) = 1.34, p = .184, Cohen’s d = 0.22; and task conflict (older: M = −1.92 ms; younger: M = 7.07 ms), t(148) = −1.68, p = .096, Cohen’s d = 0.27.

For τ, the age-group difference was not significant for response conflict (older: M = 8.57 ms; younger: M = 20.9 ms), t(148) = −0.84, p = .400, Cohen’s d = 0.14; semantic relevance (older: M = 10.4 ms; younger: M = 13.4 ms), t(148) = −0.26, p = .798, Cohen’s d = 0.04; or semantic relatedness (older: M = 0.55 ms; younger: M = 5.38 ms), t(148) = −0.39, p = .695, Cohen’s d = 0.06. Task conflict was numerically larger among older adults (M = 29.1 ms) than younger adults (M = 10.2 ms), but this difference did not reach conventional statistical significance, t(148) = 1.97, p = .0502, Cohen’s d = 0.32. Thus, despite the significant omnibus interactions for μ and σ, the only significant planned age-group difference was found for the μ component of response conflict. Given the limited number of usable trials per non-shape condition, these distributional findings should be interpreted as exploratory. The descriptive parameter estimates are presented in Table S3.

**Table S3**

***Ex-Gaussian Parameter Estimates by Age Group and Stimulus Type***

| **Age Group** | **Stimulus Type** | **N** | **μ** | **σ** | **τ** |
| --- | --- | --- | --- | --- | --- |
| **Younger Adults** | Neutral shapes | 73 | 481.2 (72.7) | 81.5 (28.0) | 143.3 (57.6) |
|  | Neutral words | 73 | 516.2 (99.9) | 88.6 (50.6) | 153.5 (69.5) |
|  | Color-associated words | 73 | 526.2 (103.0) | 87.0 (47.8) | 158.9 (70.6) |
|  | Non-response-set incongruent | 73 | 533.3 (100.5) | 96.9 (47.7) | 172.2 (74.9) |
|  | Response-set incongruent | 73 | 566.8 (127.2) | 122.7 (61.5) | 193.1 (85.7) |
| **Older Adults** | Neutral shapes | 77 | 579.1 (84.8) | 101.3 (40.2) | 143.1 (50.6) |
|  | Neutral words | 77 | 606.0 (102.4) | 99.4 (51.3) | 172.2 (78.9) |
|  | Color-associated words | 77 | 633.7 (104.8) | 109.1 (55.8) | 172.8 (67.7) |
|  | Non-response-set incongruent | 77 | 657.4 (117.4) | 122.7 (58.4) | 183.1 (75.9) |
|  | Response-set incongruent | 77 | 726.0 (155.3) | 163.1 (68.9) | 191.7 (92.5) |

***Note.*** Values are milliseconds and represent the mean of participants' fitted parameters, with standard deviations in parentheses. μ is the location of the Gaussian component, σ is the spread of the Gaussian component, and τ is the mean and standard deviation of the exponential component. N represents participants with valid estimates in all five stimulus-type conditions.
